# Activation of Functional Brain Networks in Children With Psychogenic Non-epileptic Seizures

**DOI:** 10.3389/fnhum.2020.00339

**Published:** 2020-08-25

**Authors:** Mohammadreza Radmanesh, Mahdi Jalili, Kasia Kozlowska

**Affiliations:** ^1^School of Engineering, RMIT University, Melbourne, VIC, Australia; ^2^Department of Psychological Medicine, The Children’s Hospital at Westmead, Sydney, NSW, Australia; ^3^The University of Sydney School of Medicine, Sydney, NSW, Australia; ^4^Westmead Institute for Medical Research, Sydney, NSW, Australia

**Keywords:** functional neurological disorder, functional brain networks, conversion disorder, psychogenic non-epileptic seizures, dissociative attacks, EEG

## Abstract

**Objectives:**

Psychogenic non-epileptic seizures (PNES) have been hypothesized to emerge in the context of neural networks instability. To explore this hypothesis in children, we applied a graph theory approach to examine connectivity in neural networks in the resting-state EEG in 35 children with PNES, 31 children with other functional neurological symptoms (but no PNES), and 75 healthy controls.

**Methods:**

The networks were extracted from Laplacian-transformed time series by a coherence connectivity estimation method.

**Results:**

Children with PNES (vs. controls) showed widespread changes in network metrics: increased global efficiency (gamma and beta bands), increased local efficiency (gamma band), and increased modularity (gamma and alpha bands). Compared to controls, they also had higher levels of autonomic arousal (e.g., lower heart variability); more anxiety, depression, and stress on the Depression Anxiety and Stress Scales; and more adverse childhood experiences on the Early Life Stress Questionnaire. Increases in network metrics correlated with arousal. Children with other functional neurological symptoms (but no PNES) showed scattered and less pronounced changes in network metrics.

**Conclusion:**

The results indicate that children with PNES present with increased activation of neural networks coupled with increased physiological arousal. While this shift in functional organization may confer a short-term adaptive advantage—one that facilitates neural communication and the child’s capacity to respond self-protectively in the face of stressful life events—it may also have a significant biological cost. It may predispose the child’s neural networks to periods of instability—presenting clinically as PNES—when the neural networks are faced with perturbations in energy flow or with additional demands.

## Introduction

Functional neurological disorder (FND) involves disturbances of motor and sensory function not explained by other neurological disease. Children (including adolescents) with FND present with diverse symptoms, often in combination, including loss of motor function (e.g., leg or arm paresis or weakness), loss of sensory functions (e.g., blindness, deafness, or loss of feeling in the limbs), positive movements (e.g., tremor, dystonia, or gait abnormalities), or psychogenic non-epileptic seizures (PNES). PNES are paroxysmal events that involve changes in consciousness, tone, or motor activity, and that occur in the absence of the spike-and-wave electroencephalography (EEG) pattern that is characteristic of epileptic seizures. Up to two-thirds of children with FND also suffer with complex/chronic pain, non-specific somatic symptoms—fatigue, dizziness, nausea, breathlessness—and anxiety or depression (Kozlowska et al., 2007).

Over the last decades, neuroscience studies have expanded our understanding of the neurobiology of FND. The working hypothesis from these studies is that FND symptoms emerge when stress—physical or emotional—triggers excessive activation of the brain stress systems (regions processing salience, arousal, and emotional states), which, in turn, disrupts motor- and sensory-processing regions (Blakemore et al., 2016; Kozlowska, 2017; Pick et al., 2018; Diez et al., 2020). PNES, which are paroxysmal, have been hypothesized to emerge in the context of neural network instability (Knyazeva et al., 2011; Barzegaran et al., 2012, 2016; Szaflarski and LaFrance, 2018). Potential mechanisms include a temporary disruption in neural networks, thereby compromising the horizontal and vertical integration of brain function and causing a disconnect between cortical and subcortical systems (Barzegaran et al., 2016; Kozlowska et al., 2018a), and alternatively, a time-limited increase in functional connectivity between limbic and motor regions that enables “upstream control and modulation of motor activity” (p. 213) (Li et al., 2015b; Szaflarski and LaFrance, 2018).

In the research setting, analysis of data recorded from multichannel EEG in the resting-state condition provides a window for looking at what McCraty and Childre refer to as *physiological coherence* of neural networks: “the degree of order, harmony, and stability in the various rhythmic activities within living systems over any given time period,” including “efficient energy utilization” (McCraty and Childre, 2010; Plankar et al., 2013). Researchers have also used various other terms to refer to coherence—or loss of coherence—in neural networks, including *neural synchrony* (Knyazeva et al., 2011; Li et al., 2015a), *functional connectivity* (Barzegaran et al., 2016), *loss of horizontal and vertical integration* (Lanius, 2014), and *dissociation* (a state in which coherence is lost) (van der Kruijs et al., 2012).

Knyazeva et al. (2011) used multivariate phase synchronization mapping to generate reconstructions of the whole-head topography of synchronization and looked at horizontal coherence between cortical regions in adult patients with PNES (Knyazeva et al., 2011). They found right prefrontal slowing and patchy changes in synchronization across all frequency bands. There was hypersynchronization over the left fronto-temporal, parieto-temporal, and central networks, along with hyposynchronization over neighborhood synchronization in both right and left frontal regions. The frequency of PNES increased as synchronization in frontal and parietal locations decreased. In a later study, Barzegaran et al. (2016) used the same methodology to look at vertical coherence—what they termed lagged functional connectivity—between cortical and subcortical regions in adult patients with PNES (Barzegaran et al., 2016). They found a decrease in lagged functional connectivity between the basal ganglia and limbic, prefrontal, temporal, parietal, and occipital regions, in the alpha band.

Barzegaran et al. (2012) applied a graph theoretical approach (van Straaten and Stam, 2013) to look at the function of cortical networks in adult patients with PNES (Barzegaran et al., 2012). The networks were extracted from Laplacian-transformed time-series by a cross-correlation method yielding clustering coefficient, modularity, global efficiency, and assortativity and small-worldness metrics. The *clustering coefficient* is a measure of the local connectedness of a network, and *modularity* is a measure of network hierarchy. Both metrics determine functional segregation in the brain—that is, the ability to locally process information in parallel processing streams. *Global efficiency* refers to the ability of a network to integrate information. The *assortativity* coefficient measures the correlation of node degrees in a network (Newman, 2002), an indicator of network resilience (Jalili, 2015). *Small-worldness* refers to an interplay between local and global connections and how they are different with random networks. While patients with PNES showed close to normal local and global connectivity and small-world structure, the number of PNES events per month correlated with a weakness of local connectedness and a skewed balance between local and global connectedness (quantified with small-worldness in the alpha band) and above-normal resiliency (quantified with assortativity coefficient in the beta band).

Using the same graph theoretical methodology, Xue et al. (2013) calculated clustering coefficients and global efficiency to look at coherence in neural networks in adult patients with PNES (Xue et al., 2013). Clustering coefficients (local connectedness) and global efficiency were smaller in all four frequency bands, but this difference was statistically significant only in the gamma band. Analysis of network topology in the gamma band also revealed that patients with PNES had decreased long linkage between the frontal region and posterior brain areas compared with controls. The authors discuss how gamma synchronization is a local phenomenon—either within one area or between monosynaptically coupled neurons in different areas—and that it is an important neurophysiological mechanism underlying binding, attention, and consciousness.

Umesh et al. (2017) looked at spectral power and lagged phase synchronization in the gamma band using sLORETA software in adolescents with PNES (Umesh et al., 2017). *Lagged phase synchronization* is a measure of connectivity between two signals—also known as bivariate synchronization (Knyazeva et al., 2011; Imperatori et al., 2014). On the spectral power analysis, gamma power was higher in the left parietal regions and lower in the right temporal region. In addition, decreased gamma band current source density was found in the right posterior parietal cortex, posterior cingulate cortex, and superior temporal gyrus. Connectivity analysis showed a reduced intracortical lagged coherence between right posterior cingulate gyrus and right middle temporal gyrus (trend level only).

Meppelink et al. (2017) recorded EEG spectral power changes in three adult patients with PNES (and patients with epilepsy) prior to seizure events (Meppelink et al., 2017). Patients with PNES—but not those with epilepsy—showed a decrease in beta power (desynchronization) prior to the PNES (minimum beta power at 5–6 s prior to the PNES).

Arikan et al. (2020) use quantitative EEG (QEEG) and fast Fourier transformation to examine spectral power across all frequency bands in adults with PNES. They found increased power at the following bands and locations: C3-high beta (25–30 Hz), C3-gamma (30–80 Hz), C3-gamma-1 (31–40 Hz), C3-gamma-2 (41–50 Hz), P3-gamma (30–80 Hz), P3 gamma-1 (31–40 Hz).

Finally, emerging evidence from neuroimaging studies in adult patients with PNES—reviewed by Szaflarski and LaFrance (2018)—also suggests that neural network instability may be involved in PNES generation and maintenance (Szaflarski and LaFrance, 2018).

Taken together, the studies summarized above suggest that the neural networks of patients with PNES, when compared to those of healthy controls, may be less resilient in the face of additional demands and more prone to temporary states of disorder or aberrant changes in functional connectivity—resulting in PNES.

The aim of the present study—the first study conducted with younger children—was to examine whether children with PNES show alterations in neural network function. The study utilized graph theory to examine eyes-closed, resting-state EEG data recorded from children presenting with PNES (PNES group), those presenting with other FND symptoms (FND-other group), and healthy controls. In the light of previous studies—discussed above—we hypothesized that children with PNES would demonstrate a shift away from optimal function on one or more network parameters of neural network function. Based on a study by Umesh et al. (2017) with adolescents—the only study with adolescent participants with PNES—we hypothesized that changes in network metrics would be found in high frequency bands (gamma and beta). The secondary goal was to examine whether aberrant network parameters correlated with arousal.

## Materials and Methods

### Participants

Seventy-nine children and adolescents with FND were recruited between August 16, 2006, and August 16, 2016, from the consultation-liaison psychiatry team at a tertiary-care pediatric hospital in New South Wales, Australia, to undergo a research EEG in the rest-eyes-closed condition. The EEG was part of a standardized laboratory assessment: one component of a broader FND research program. The diagnosis of FND, using modified DSM-IV-TR criteria (American Psychiatric Association, 2000), was made by a pediatric neurologist following a comprehensive neurology assessment. For the group of children who presented with seizure events and who received a diagnosis of PNES, the neurology assessment included a clinical video EEG—the gold standard for the diagnosis of PNES—which enabled the neurologist to observe the seizure events and EEG tracing concurrently. After the EEG had been read and reported, the diagnosis was subsequently confirmed by a child and adolescent psychiatrist following a psychiatric assessment (Kozlowska et al., 2013b). Functional impairment was recorded using the Global Assessment of Functioning (GAF). Consistent with later-adopted DSM-5 criteria, the study did not adhere to the DSM-IV-TR “psychological stressor criterion,” because previous epidemiological research with children/adolescents had highlighted that the psychological-stressor criterion was too narrow and that physical stressors (e.g., antecedent illness or injury) were common (Kozlowska et al., 2007). Instead, all antecedent stressors reported by the child and family at assessment—both psychological and physical—were documented. Also consistent with DSM-5 criteria, all participants with motor symptoms had documented positive signs on neurological examination, including the worsening of symptoms with attention and a decrease of symptoms with distraction. Following the psychiatric assessment—and depending on the level of functional impairment—patients were offered either outpatient or inpatient treatment (specifically, admission to the hospital’s Mind-Body Program for treating functional somatic symptoms) (Kozlowska et al., 2012). Subjects with developmental delay were excluded from the study because the laboratory assessment required that the child be independently able to follow a series of instructions depicted on a computer screen.

Seventy-nine healthy controls, age- and sex-matched, were selected from the Brain Resource database from the same catchment area, a database developed for neuroscience research. Control participants were screened for the absence of mental health disorders, history of head injury, family history of mental health disorders, and chronic health concerns. To meet inclusion criteria, all healthy controls would have been scored into the upper two brackets on the GAF (score ≥ 81).

The study was approved by the Sydney Children’s Hospital Network Ethics Committee and Sydney West Area Health Service Human Research Ethics Committee. Participants and their legal guardians provided written informed consent.

### Procedure

Participants with FND attended testing in the laboratory soon after the clinical assessment while they were experiencing functional neurological symptoms or during a period of time in which their PNES episodes were occurring. Participants were asked to rest quietly, with their eyes closed for 3 min while the EEG, along with concurrent electrocardiogram (ECG) data, was recorded. As part of the laboratory protocol, participants also completed the Spot-the-Word Test (Intelligence Quotient estimate), the depression, anxiety, and stress scales (DASS), and the early life stress questionnaire (ELSQ). For details about the GAF measure and self-report questionnaires, see Kozlowska et al. (2017a).

All controls had completed the same test battery as the clinical sample.

### EEG Acquisition Methodology and Preprocessing

EEG was recorded using 26 cephalic sites at Fp1, Fp2, Fz, F3, F4, F7, F8, Cz, C3, C4, FC3, FCz, FC4, T3, T4, T5, T6, Pz, P3, P4, O1, O2, and Oz electrode sites (10–20 International System). A QuickCap (Neuroscan) was used to acquire EEG data from these cephalic sites. EEG data were referenced to the average of A1 and A2 (mastoid) electrodes sites. For the horizontal electrooculogram (EOG), horizontal eye movements were recorded with electrodes placed 1.5 cm lateral to the outer canthus of each eye. Vertical eye movements were recorded with electrodes placed 3 mm above the middle of the left eyebrow and 1.5 cm below the middle of the left bottom eyelid. Skin resistance was kept at < 5 kΩ. Scalp, EOG, and other potentials were amplified and digitized continuously by a system (NuAmps, SCAN 4.3) having a frequency response from DC to 100 Hz (above which, attenuating by 40 dB per decade), a sampling rate of 500 Hz, and a 22-bit resolution digitization. Correction for eye-blink artifact was carried out on the 26 cephalic sites using a technique based on Gratton et al. (1983) using the recorded EOG data. The Brain Resource database software was used to perform artifact handling on the EEG. Epochs are rejected if the signal at three or more sites exceeded 100 μV voltage swing for that particular epoch.

For details about the ECG methodology to obtain heart rate variability and heart rate, see Kozlowska et al. (2015a). For ECG data, outliers > 2 standard deviations were removed from the FND and control samples.

### Constructing Brain Functional Networks

In order to construct EEG-based functional brain networks, we first assigned a node to each EEG electrode. Then, for each subject, coherence values of time series data were calculated epoch-wise and averaged over epochs. These coherences are collapsed for the frequency range corresponding to each band: 30–70 Hz for gamma (γ); 13–30 Hz for beta (β); 7–13 Hz for alpha (α); 3–7 Hz for theta (θ); and 1–3 Hz for delta (δ). This way, we could construct a weighted 28 × 28 coherence matrix for any of the frequency bands of each subject. The obtained weighted coherence matrices cannot be used, however, to calculate network metrics; the matrices need to be properly binarized. To binarize a weighted connectivity matrix, one approach is to specify a threshold and to set all values above this threshold to 1 and otherwise 0. The constructed binary networks might not be comparable, however, since they may have different numbers of links—that is, different densities. It is well known that many network metrics are heavily dependent on the network density. An alternative approach is therefore to binarize the connectivity matrices in a way that they produced networks having the same density (Achard and Bullmore, 2007; Tahaei et al., 2012). We binarized the weighted matrices in separate calculations for density values ranging from 0.05 to 0.3 with 0.01 intervals. Finally, some neurobiologically meaningful graph theory metrics were computed based on these binary networks.

### Graph Theory Metrics

We examined global efficiency, local efficiency, and modularity metrics. *Global efficiency* is a measure of network performance based on the global topology of the network. Global efficiency measures distant information flow in a network (Latora and Marchiori, 2001). It often increases by increasing network density (i.e., number of connections); inversely related to topological distance between nodes, it measures global information exchange across a network (Latora and Marchiori, 2001). Global efficiency is defined as the inverse of the average characteristic path length among all nodes. Formally, it is calculated as

$$E_{g\,l\,o\,b}=\frac{1}{N\,\left(N-1\right)}\,\sum_{i,j}\frac{1}{L_{i,j}}$$

where *N* is the network size and *L*_*i,j*_ is the average distance between node *i* and *j* in the network. In functional brain networks, global efficiency measures the integrated information processing among distributed parts of the network and the overall network capacity to transfer information in parallel (Bullmore and Sporns, 2012).

*Local efficiency* measures the average efficiency of information flow within local sub- neighborhoods and is defined as the inverse of the shortest average path length of all neighbors of a given node among themselves. Formally, it is calculated as

$$E_{l\,o\,c\,a\,l}=\frac{1}{N_{S\,N_{i}}\,\left(N_{S\,N_{i}}-1\right)}\,\sum_{j,k\in S\,N_{i}}\frac{1}{L_{j,k}}$$

where *N*_*SN_i*_ is size of the sub-network *SN*_*i*_. Local efficiency describes how effectively information is transferred within the first neighbors of node *i*, when node *i* is removed from the network. It provides a basis for information segregation process in the network (Rubinov and Sporns, 2010). Global and local efficiencies scale in the range 0–1, with a value of 1 indicating maximum efficiency.

It has been frequently shown that many real networks are clustered into different modules, where intra-module connections are dense while inter-modular links are sparse (Girvan and Newman, 2002). Characterization of modular structures as basic “building blocks” in the brain network provides insights into the brain’s tendency to segregate into relatively independent and local neighborhoods. *Modularity index* has been proposed in the literature to measure level of modular structure in a network. It is calculated as

$$Q=\sum_{k}\left[e_{i\,i}-{\left(\sum_{j\in k}e_{i\,j}\right)}^{2}\right]$$

where *k* is the predefined number of modules and *e*_*ij*_ is the portion of all links connecting nodes in module *i* with those in module *j*. Higher values of *Q* correspond to higher level of modular structure.

### Statistical Assessment of Graph Theory Metrics

The Wilcoxon rank-sum test was used to examine for statistically significant differences (*p* < 0.05) between the PNES group and controls and between the FND-other group and controls.

### Correlations of Graph Theory Metrics and Arousal

Spearman rank correlation was used to investigate the relationship between arousal—measured by RMSSD-HRV (root mean squared successive differences of the interbeat intervals, the time domain component of heart rate variability measured in ms^2^)—and graph theory metrics in the PNES group and FND-other group.

### Analysis of ECG, Self-Report Data, and Data Gathered on Clinical Assessment

Chi-square analyses (for categorical variables) and independent *t*-tests (for continuous, normally distributed data) were used to calculate differences between the entire FND cohort and control group and between the PNES subgroup and FND-other subgroup. For the analysis of GAF scores, healthy controls were assigned an arbitrary value of 81, lowest value in the healthy range.

### *Post hoc* Examination Pertaining to the Potential Role of Comorbid Anxiety and Depression on Graph Theory Metrics

Because anxiety and depression are common comorbid diagnoses in children with PNES and FND-other, the potential role of comorbid anxiety and depression was examined *post hoc*. *T*-tests and Chi Square analyses were used to compare the PNES and FND-other groups regarding rates of anxiety/depression, as determined by the DASS and by clinical diagnosis (DSM-IV-TR), respectively. Spearman rank correlation was used to investigate how graphic theory metrics was related to the DASS score and clinical diagnosis in the PNES group.

### *Post hoc* Comparison of Network Metrics Between the PNES and FND-Other Groups

Because the visual representation of results suggested that changes in graph theory metrics in the PNES and FND-other group reflect changes along a spectrum, a comparison between the PNES and FND-other group was run *post hoc*.

## Results

### Examination of the Data

Examination of the data revealed adequate EEG data sets for 66/79 (83.5%) participants with FND (including both PNES and FND-other) and 75/79 (94.93%) healthy controls. Missing EEG data (16 and 5%, respectively) was due to a general technical failure in three participants with FND and to the failure of at least one electrode during EEG data recording in the remaining participants and controls whose data were inadequate. For the FND and control groups respectively, missing data for the other measures was as follows: ECG (6%, 8%); Spot-the-Word Test (10.6, 33%); DASS (19%, 0%); and ELSQ (21%, 0%). ECG data were normally distributed.

### Final Sample: Participant and Control Characteristics

The final FND sample (both PNES and FND-other) comprised 66 children/adolescents (47 girls and 19 boys) aged 8.43–18.16 years (mean = 13.48; *SD* = 2.15; median = 13.58) and 75 sex- and age- matched healthy controls (53 girls and 22 boys) aged 8.33–17.99 years (mean = 13.54; *SD* = 2.38; median = 13.38) (see Tables 1, 2). There were no differences on sex, age, and estimates of IQ between the FND and healthy control groups (see Table 1). In line with previous studies, in comparison to controls, children with FND had higher levels of autonomic arousal (lower RMSSD-HRV and higher [HR]), had higher levels of anxiety, depression and stress on the DASS, and reported more adverse childhood experiences (ACEs) on the ELSQ (see Table 1). Differences between the PNES and FND-other group were found on two parameters (see Table 3). Children with PNES and their families reported maltreatment—including exposure to domestic violence—more frequently than children with FND-other on clinical assessment (18/35 PNES vs. 5/31 FND-other) (see Table 2). On the Spot-the-Word test (an estimate of IQ)—on which the children with PNES performed less well than children with other FND symptoms (see Table 3).

**TABLE 1:** Comparisons between FND and healthy-control group on demographics, IQ, ECG measures, and self-report.

**Demographic/Measure**	**FND group mean value/total score**	**Healthy-control group mean value/total score**	**All FND vs. Controls *t*/**χ^2^ **(*p*)**
Age	13.48	13.54	−0.168 (0.867)
Sex	47/66 girls	53/75 girls	0.0051 (0.943)
Spot-the-word Test (IQ estimate)	38.85	39.38	0.407 (0.685)
RMSSD-HRV (arousal estimate)	55.51	68.73	−2.69 (0.008)
Heart rate (arousal estimate)	85.16	75.47	5.30 (<0.001)
DASS Total Score	27.83	7.57	6.08 (<0.001)
ELSQ (adverse childhood event load)	4.36	1.39	4.41 (<0.001)
GAF	40.22 SD 13.43, range, 11–75	Assigned an arbitrary value of 81 (lowest value in the healthy range)	−24.66 (<0.001)

**TABLE 2:** Clinical information about participants with FND obtained from the clinical assessment.

**Clinical information, diagnoses and comorbid symptoms for all children with FND (PNES and FND-Other)**	**Number (*n* = 66)/Mean/Median/Range**	**Percentage**
Illness duration (months)	Mean 4 Range 2 days to 36 months	
Total number of FND symptoms	Mean 2.38, median 2 range: 1–7	
Comorbid Pain	47	71.2%
Any non-specific somatic symptom	45	68%
Comorbid fatigue	27	41%
Dizziness	25	38%
Breathlessness	16	24%
Nausea	16	24%
**Medical conditions**		
Epilepsy	2	3%
Asthma	2	3%
Glomerulonephritis	1	1.5%
Cerebral palsy	1	1.5%
Migraine	1	1.5%
Congenital heart disease	1	1.5%
Cancer in the kidney (operated on)	1	1.5%
Hernia (operated on)	1	1.5%
**Children meeting criteria for a mental health diagnosis**		
Anxiety disorder (DSM-IV-TR)	31	47%
Depressive disorder (DSM-IV-TR)	10	15%
Mixed anxiety and depression (DSM-IV-TR)	8	12%
Amnesia or other dissociative symptoms	16	24%
**Antecedent life events (maltreatment-related events are denoted by an asterisk)**		
Total adverse childhood life events (ACEs) elicited on clinical assessment (including maltreatment events)	Mean 5.2 Median 5 Range 1–10	
Family conflict	41	62%
Child physical illness	33	50%
Bullying	32	49%
Maternal mental illness	27	41%
Loss via separation from a loved one or a close friend	24	36%
Loss via death of a loved one	22	33%
Maternal physical illness	16	24%
Paternal mental illness	15	23%
Paternal physical illness	13	20%
Moving house that had been stressful	13	20%
Migration	3	5%
Maltreatment any	23	16%
Exposure to domestic violence	14	21%
Physical abuse	9	14%
Neglect	8	12%
Sexual abuse	7	11%
**Intelligence quota estimated from school testing and school reports**		
Superior range (120+)	13	19.7%
Average range (80–119)	50	75.8%
Borderline range (70–79)	3	4.5%

**TABLE 3:** Comparisons between PNES and FND-other groups on demographics, IQ, ECG measures, self-report, and clinical data.

**Demographics**	**PNES group: Number (% of *n* = 35)/Mean/Median/Range**	**FND-other group: Number (% of *n* = 31)/Mean/Median/Range**	**PNES group vs. FND-other group*t*/χ^2^ (p)**
Age	Mean 13.77 Median 14.14 Range 9.29–18.16	Mean 13.15 Median 13.21 Range 7.72–16.15	1.182 (0.241)
Sex	Boys 11 (31%) Girls 24 (69%)	Boys 8 (26%) Girls 23 (74%)	0.254 (0.614)
**Data from laboratory assessment**			
Spot-the-Word Test (IQ estimate)	Mean 38.28	Mean 41.70	**−2.290 (0.026)^a^**
RMSSD-HRV (arousal estimate)	Mean 35.06	Mean 36.07	1.185 (0.241)
Heart rate (arousal estimate)	Mean 83.18	Mean 86.06	−1.154 (0.253)
DASS Total Score	Mean 27.24	Mean 24.13	0.538 (0.593)
ELSQ (adverse childhood event load)	Mean 5.00	Mean 3.67	1.152 (0.255)
**Data from clinical assessment**			
GAF	Mean 39.14 Range 15–65	Mean 41.45 Range 11–75	-0.694 (0.490)
Illness duration (months)	Mean 4.41 months Range 3 days to 24 months	Mean 5.24 months Range 2 days to 36 months	−0.493 (0.624)
Total number of FND symptoms	Mean 2.66, Median 2 Range 1–7	Mean 2.16 Median 2 Range: 1–5	1.432 (0.157)
Imaging (CT or MRI) as part of clinical assessment	27 (77.1%)	27 (87.1%)	1.094 (0.295)
Number of children taking medication	4 (1.1%)	4 (1.3%)	0.026 (0.871)
Comorbid pain	22 (62%)	25 (81%)	2.537 (0.111)
Any non-specific somatic symptom (fatigue, dizziness, breathlessness, nausea)	Mean 1.37 Median 1 Range 0–4	Mean 1.16 Median 1 Range 0–4	0.709 (0.481)
Children meeting criteria for a mental health diagnosis of anxiety or depression or mixed anxiety/depression	26 (74.3)	23 (74.2)	0.00001 (0.993)
Total adverse childhood life events (ACEs) elicited on clinical assessment (including maltreatment events)	Mean 5.40 Median 6 Range 1–10	Mean 4.97 Median 5 Range 1–10	0.754 (0.453)
Maltreatment events elicited on clinical assessment	Mean 0.89 Median 1 Range 0–4	Mean 0.23 Median 0 Range 0–2	**3.169 (0.003)**
Differences in IQ quotients estimated from school testing and school reports [superior (120+); average (80–119); borderline (70–79)]	Superior 6 (17%) Average 27 (77%) Borderline 2 (6%)	Superior 7 (23%) Average 23 (74%) Borderline 1 (3%)	0.500 (0.783)

*^*a*^Figures in bold are statistically significant.*

**FIGURE 1:**
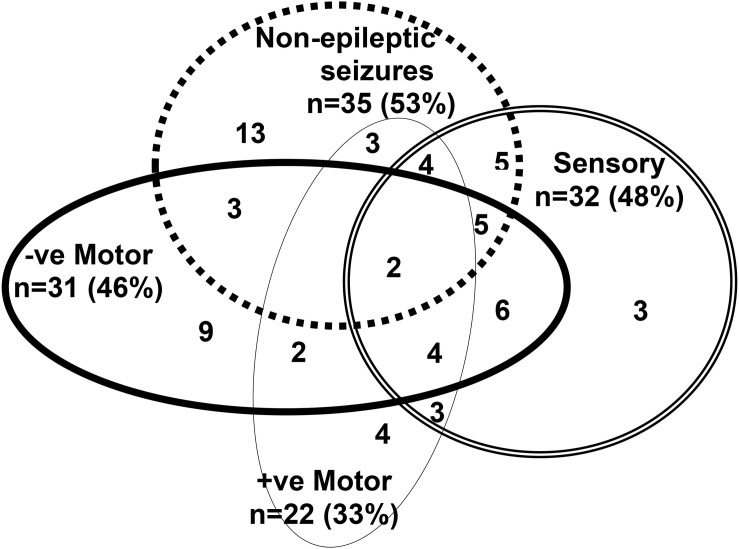
This figure depicts the functional neurological symptoms experienced by the 66 children with FND who were included in the analysis. Thirty five children and adolescents presented with psychogenic non-epileptic seizures (PNES) as part of their presentation (top dotted circle). PNES presented as follows: paroxysmal limb movements (*n* = 9); syncope-like events (*n* = 7); paroxysmal limb movements and syncope-like events (*n* = 6); paroxysmal limb movement and spacing-out events (*n* = 5); paroxysmal limb movements, syncope-like events, and spacing-out events (*n* = 3); syncope-like events and spacing out (*n* = 3); paroxysmal limb movements and incontinence (*n* = 1); and syncope-like, spacing-out events, choking sensation, and paresthesia in the fingers (*n* = 1). Thirty- one children presented with other functional neurological symptoms, but no PNES. Across both groups—PNES and FND-other—other functional neurological symptoms were mixed and occurred in various combinations: leg weakness/paralysis, loss of speech (negative motor symptoms); functional tics/tremor/jerking, dystonia in a limb, cough, vomiting/rumination, astasia-abasia (positive motor symptoms); and leg anesthesia/paresthesia, loss of vision, loss of hearing, globus sensation (sensory symptoms).

Participants (PNES and FND-other) presented with one or more functional neurological symptoms (see Figure 1) and significant functional impairment (see GAF score in Tables 1, 3). Illness duration was 2 days to 36 months (mean = 4 months). Thirty-five presented with PNES—with or without other motor or sensory symptoms—and the other 31 presented with other motor or sensory functional neurological symptoms (but no PNES) (see Figure 1). Comorbid pain (47/66; 71.2%) and non-specific somatic symptoms (45/66; 68.2%)—including fatigue, dizziness, nausea, and breathlessness—were reported by more than two-thirds of participants (see Table 2). Eighty percent (53/66) of participants met criteria for comorbid mental health diagnoses, with anxiety being the most common (see Table 2). Ninety-four percent (62/66) of participants had symptoms that were sufficiently disabling—for example, their mobility or self-care skills were severely compromised, or they were, because of their symptoms, unable to attend school—to be offered treatment in the inpatient, hospital-based Mind-Body Program. Participants’ families spanned all socioeconomic levels: professional (*n* = 24; 36%), white collar (*n* = 25; 38%), blue collar (*n* = 14; 21.2%), and unemployed (*n* = 3; 5%).

Most of the participants had been healthy prior to the onset of their functional neurological symptoms (see Table 2 for medical diagnoses). At the time of testing, 2 participants with comorbid epilepsy and one who had been misdiagnosed as having epilepsy were taking anti-epileptic medication (sodium valproate [*n* = 2] and sodium valproate and levetiracetam [*n* = 1]), 4 with comorbid pain were taking gabapentin (*n* = 2) or amitriptyline (*n* = 2), and one with comorbid depression and sleep problems was medicated with fluoxetine, clonidine, and melatonin. Children taking medications were equally split across the PNES and FND-other groups (see Table 3).

### Results From Graph Theory Metrics

**FIGURE 2:**
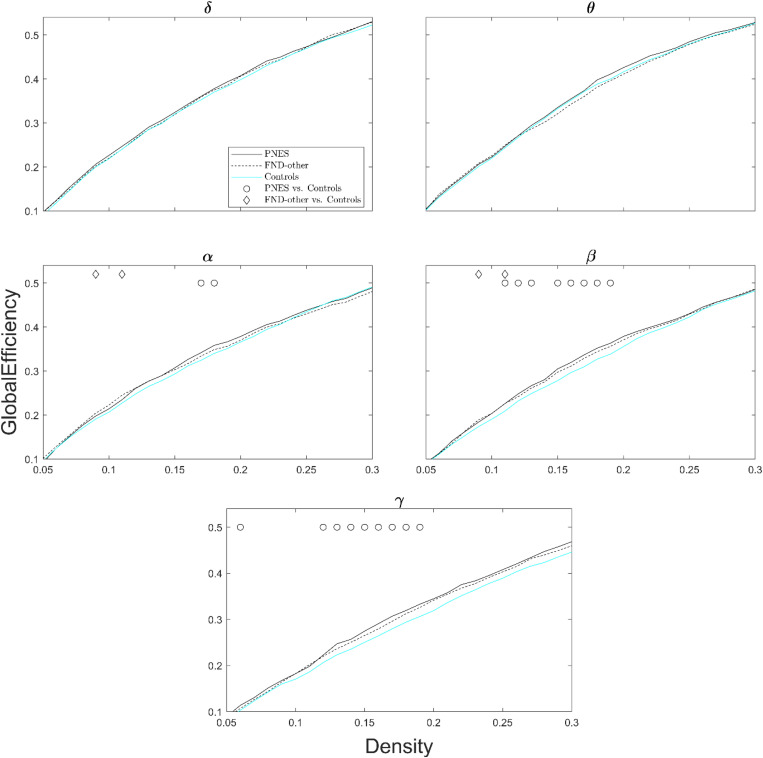
Global efficiency as a function of network density for patients with PNES, patients with other FND symptoms and controls. The plots show the global efficiency of functional brain networks as a function of density in the PNES group (black lines), FND-other group (black dash lines) and controls (cyan lines) for different frequency bands from 0.05 to 0.3. Circle and diamond shapes indicate the densities that the differences in PNES/FND-other vs. controls is less than *P* < 0.05 (Wilcoxon’s ranksum test; uncorrected for multiple comparison).

Figure 2 shows global efficiency for all three groups (PNES, FND-other, and healthy controls) as a function of network density across all frequency bands. The PNES group showed increased global efficiency compared to healthy controls for a range of medium densities in the gamma and beta bands. There were some patchy differences in the alpha band and no significant differences in theta and delta bands. Children in the FND-other group showed scattered increases in global efficiency in the beta and alpha bands.

**FIGURE 3:**
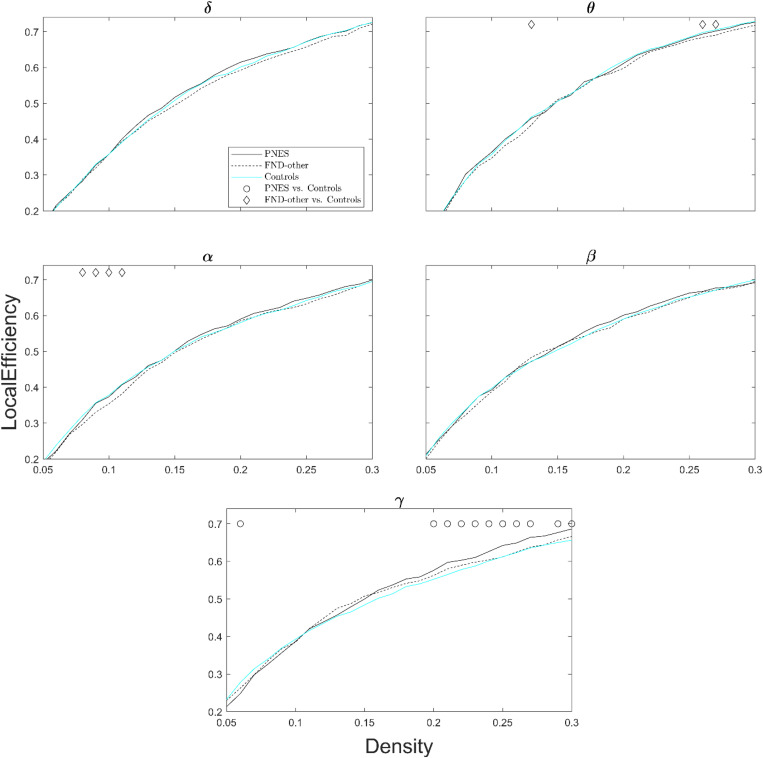
Local efficiency as a function of network density for patients with PNES, patients with other FND symptoms and controls. The plots show the local efficiency of functional brain networks as a function of density in the PNES group (black lines), FND-other group (black dash lines) and controls (cyan lines) for different frequency bands. Other designations are as Figure 2.

Figure 3 shows local efficiency as a function of network density across all frequency bands. The PNES group showed increased local efficiency for medium- to high-density values in the gamma band. Children in the FND-other group showed scattered increases in local efficiency in the alpha and theta bands.

**FIGURE 4:**
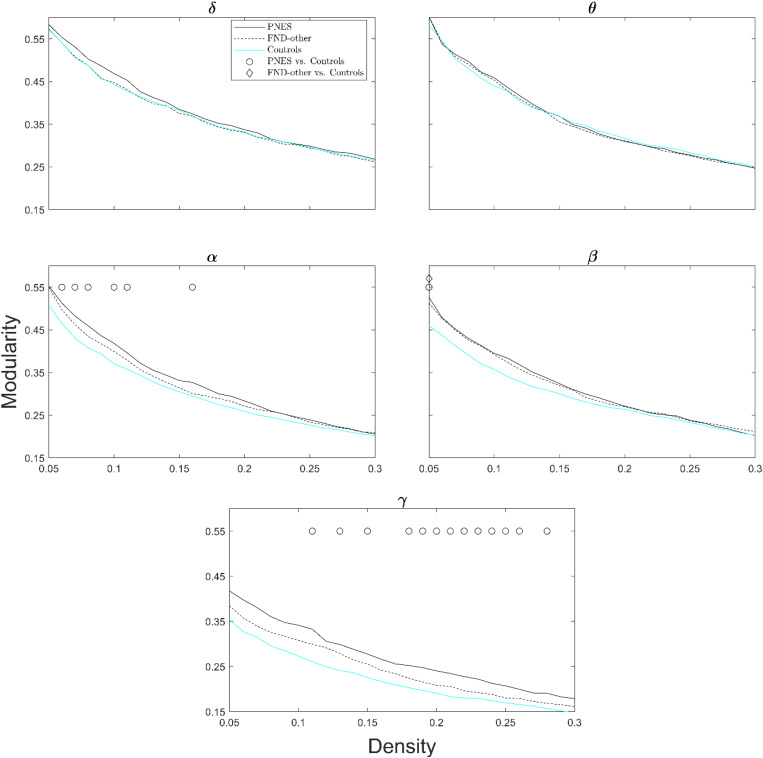
Modularity index as a function of network density for patients with PNES, patients with other FND symptoms and controls. The plots show the modularity of functional brain networks as a function of density in the PNES group (black lines), FND-other group (black dash lines) and controls (cyan lines) for different frequency bands. Other designations are as Figure 2.

Figure 4 shows modularity index as a function of network density across all frequency bands. The PNES group showed increased modularity for broad range of densities in the gamma and alpha bands. Children in the FND-other group showed no significant differences in modularity.

**FIGURE 5:**
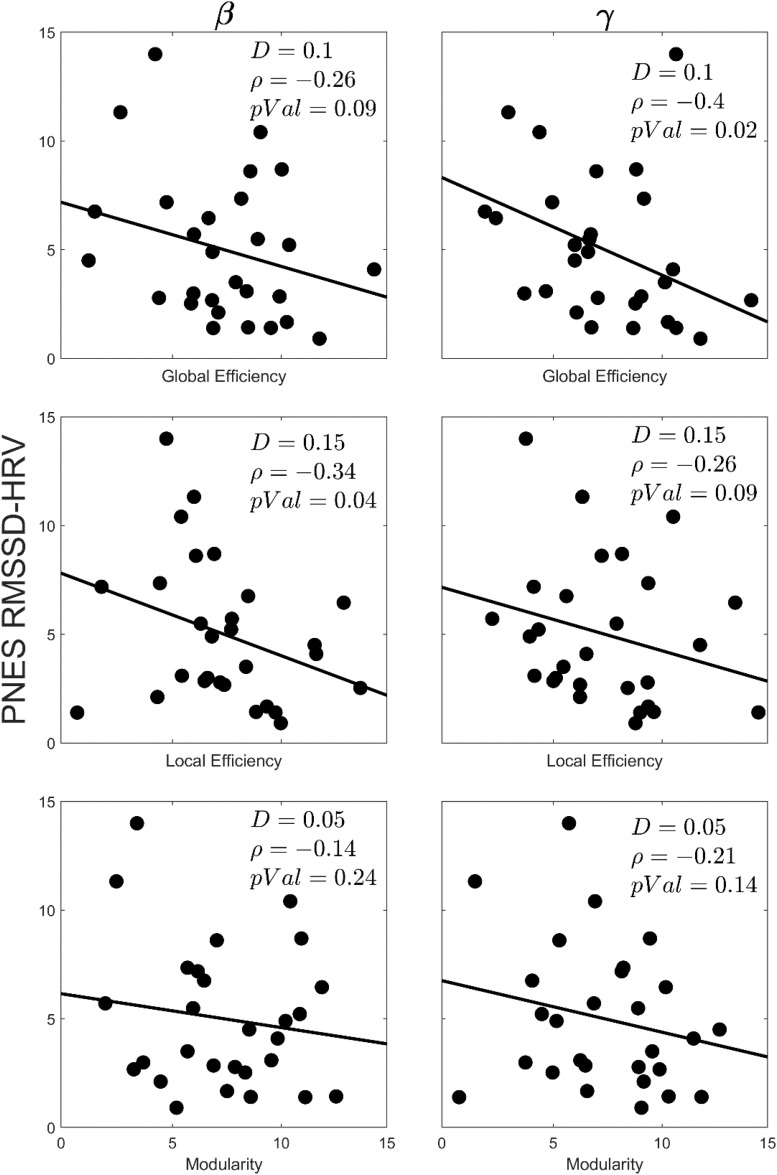
Scatterplots for correlations between the graph theory metrics and the RMSSD-HRV obtained for EEG beta and gamma bands in children with PNES. The network density, correlation value and *p*-value for each plot has been indicated by *D*, ρ, and *pVal*, respectively.

RMSSD-HRV is a sensitive measure of arousal; as arousal increases, the RMSSD-HRV value decreases. Figure 5 presents scatter plots of the correlations between the graph theory metrics (viz., global efficiency, local efficiency, and modularity) and RMSSD-HRV for the PNES group in select density values. The negative correlation values for all metrics reveals that the lower the value of RMSSD-HRV (the higher the arousal), the higher the value of the network metric. The correlations were significant (*p* < 0.05; one sided) in global efficiency (gamma band) and local efficiency (beta band).

In the FND-other group there was no correlation between RMSSD-HRV and graph theory metrics in select density values.

The PNES and FND-other groups were well matched in terms of the percentage of children who experienced anxiety and depression as determined both by the DASS and by clinical diagnosis (DSM-IV-TR) (see Table 3). In the PNES group there was no correlation between graph theory metrics and either DASS scores or clinical diagnosis in select density values.

Because the visual representation of results suggested that changes in graph theory metrics in the PNES group (widespread changes) and FND-other (patchy changes) occurred along a spectrum, a comparison between the PNES and FND-other group was run *post hoc*. On all graph theory metrics, the differences between groups were not significant.

## Discussion

In the present resting-state EEG study, we used graph theory to examine neural network function in children presenting with PNES, children presenting with other functional neurological symptoms (but no PNES), and healthy age- and sex-matched controls. Compared to controls, children in the PNES group showed widespread changes in network metrics: increased global efficiency (gamma and beta bands), increased local efficiency (gamma band), and increased modularity (gamma and alpha bands). In children with PNES, increases in network metrics—global efficiency in the gamma band and local efficiency in the beta band—correlated with arousal; the higher the arousal, the greater the increase of the network metric. Our interpretation of these findings is that in the context of high arousal, the neural networks of children with PNES showed, in higher-frequency bands, enhanced capacity for distant information flow (global efficiency), enhanced capacity for information flow within local sub-neighborhoods (local efficiency), and increased efficiency for local processing of information in parallel-processing streams (modularity). Children in the FND-other group showed scattered and less-pronounced changes in network metrics—increased global efficiency (beta and alpha bands) and increased local efficiency (alpha and theta bands)—and no correlation between graph theory metrics and arousal.

In the PNES group, increases in values in the gamma band in medium- to high-density values was found across network metrics. The gamma band reflects fast cortical oscillatory activity—the coordinated activity of large numbers of neurons—that occurs during conscious perception (Jensen et al., 2007; Malik and Amin, 2017). Converging evidence from animal and human research suggests that synchronization in the gamma band is important for neural communication and, in particular, for neural network processes that underpin attention, working memory, and long-term memory (Jensen et al., 2007). In addition, prominent gamma waves are associated with states of high arousal, anxiety, and stress (Abhang et al., 2016).

In the current study, arousal, anxiety, and exposure to stressful live events were documented by low RMSSD-HRV, DASS score, and ELSQ score, respectively. Since “evolution has designed high arousal states to be functional” (p. 430) (Hanoch and Vitouch, 2004)—enabling the organism to organize self-protectively to manage the stressful challenge—the raised network metrics in the gamma band in children with PNES may reflect a brain-wide hypervigilance response. Hypervigilance is a state of increased readiness that emerges in the context of threat (or perceived threat), where attention is directed to events tagged as salient, and where reflexive functions with survival value are prioritized over cognitive-processing functions. This shift in processing has been documented in previous studies from FND research program with children with FND—including those with PNES. In studies measuring reaction times, children with FND (vs. healthy controls) showed faster reaction times to emotion faces (Kozlowska et al., 2013a; Kozlowska, 2016) and slower reaction times to cognitive tasks on the IntegNeuro test battery (Kozlowska et al., 2015b). On formal structured assessments of attachment, children with FND demonstrated a higher rate of linguistic markers for trauma- and loss- memories (salient memories for past threat) (Kozlowska et al., 2011), and on formal cognitive testing they showed decreased working-memory capacity for non-salient items on multiple subtests (Kozlowska et al., 2015b).

Another key finding emerging from this study is that changes in network metrics were more pronounced in children whose FND presentation included PNES than in children with no PNES. Children in the FND-other group showed only a patchy increases in global and local efficiency (at lower frequency bands), and these changes did not correlate with arousal. In general, neurophysiological responses lie on a normative curve, where the individuals on the right-hand end of the curve—due to genetics or epigenetic priming mechanisms—respond to challenges with a more robust, or even an exaggerated, response. The findings of this study suggest that children with PNES show a particularly robust neural network response to threat characterized by activation of neural networks in the gamma band. In the short term, this activation in neural networks is likely to confer an adaptive advantage, one that facilitates neural communication and the child’s capacity to respond self-protectively in the face of stressful life events. In the longer term, however, all stress-related adaptions have a biological cost—known as allostatic load (McEwen, 1998)—which is discussed below.

The lack of significant findings in the *post hoc* comparison between the PNES group and FND-other group—together with the positive correlation between graph theory metrics and arousal in the PNES group—suggests that the changes in graph theory metrics in children with FND lie on a spectrum as a function of arousal. This finding is of particular interest to clinicians. In clinical practice it is not uncommon for a child to present with FND-other symptoms, and for the presentation to then change, over time, to include PNES, presumably in relation to increasing arousal [see Gray et al. (2020) or Kozlowska (2020, forthcoming) for case vignette examples].

Working from a systems perspective (Capra, 1997), the brain is conceptualized as a self-organizing system whose functions emerge from complex, non-linear dynamics within neural networks (Pezard and Nandrino, 2001; Uhlhaas and Singer, 2015; Goldbeter, 2018). As a living system, the brain is dependent on a constant flow of matter and energy, enabling it to continually regenerate itself and to maintain its stability of structure and function (Capra, 1997). The term *physiological coherence*, as mentioned earlier, denotes an overall equilibrium that is characterized by an efficient utilization of energy in the various rhythmic activities within living systems over any given time period (McCraty and Shaffer, 2015). Physiological coherence is associated with health and well-being. But the increased activation of neural networks, as seen in our patients with PNES, reflects a new level of organization that can be maintained only with additional expenditures of energy. Given that this shift away from physiological coherence is highly dependent on the flow of energy and availability of resources, it is more vulnerable to periods of instability when faced with perturbations in energy flow or additional demands—for example, in the context of new threat or stress or perceived threat or stress. By the same token, we hypothesize that when the neural networks of children with PNES reach a critical point of instability, there ensues a time-limited period of disorganization, where the brain reverts temporarily to an evolutionarily older state of organization—a process that the distinguished nineteenth-century neurologist J. Hughlings Jackson referred to as *dissolution* (Jackson, 1884). This conceptualization of PNES is similar to the conceptualization of the common faint as a non-linear, non-stationary, time-dependent event that involves a loss of healthy synchronization between cardiorespiratory parameters (Ocon et al., 2010). Interestingly, a small pilot study with three adult patients with PNES (not yet replicated) showed a decrease in beta power (desynchronization)—analogous to Jackson’s idea of dissolution—prior to the PNES (Meppelink et al., 2017).

Jackson defined *dissolution* as “a process of undevelopment; it is a ‘taking to pieces’ in the order from the least organized, from the most complex and most voluntary, toward the most organized, most simple, and most automatic” (p. 591) (Jackson, 1884). From the contemporary perspective, the state of dissolution that occurs in children with PNES is likely to reflect one or more of the following processes: (1) disruption of prefrontal cortex function and a release of subcortical motor programs that are otherwise inhibited by the prefrontal cortex; (2) disruption of prefrontal cortex/cerebellum function and a release of subcortical emotion-expression programs that are otherwise regulated by the prefrontal cortex/cerebellum (Parvizi et al., 2001; Okun et al., 2004); (3) a time-limited change in functional connectivity, one that prioritizes limbic control of motor regions and “upstream control and modulation of motor activity” (p. 213) (Szaflarski and LaFrance, 2018); (4) changes in the level of consciousness reflecting a functional disruption of the neural networks—from the brain stem to the cortex—underpinning human consciousness (Tindall, 1990); and (5) changes in musculoskeletal tone reflecting either increased activity in brain stem regions that mediate increases in muscle tone or a temporary disruption in signals from the hypothalamus and the brain stem that set muscle tone (Walker and Carrive, 2003). Because PNES may involve one or any combination of the above-described processes, semiology varies widely both between patients and in any one individual child across time and across presentations (Szabo et al., 2012).

Contemporary neuroscience research suggests a number of factors—neurophysiological mechanisms—by which child’s vulnerable neural network may be *tipped* into a temporary (paroxysmal) state of dissolution resulting in the emergence of PNES. The first factor is hyperventilation (HV), which has a biphasic effect on brain function. Initially, HV increases cortical excitation (phase 1), and if continued, it destabilizes cerebral hemodynamic and causes cerebral hypoxia (phase 2)—and a disruption of energy flow in neural networks—via hypocapnia-related cerebral vasoconstriction and the Bohr effect, depicted on the EEG as an increase in delta waves (or slowing) [for review of the literature, see Supplementary Text Box 1 in Kozlowska et al. (2017b)]. The effects of HV are more pronounced in children (Epstein et al., 1994; Yamatani et al., 1994). In a study of 60 children with PNES, Kozlowska et al. (2017b) found that approximately half of the children triggered their PNES via HV (Kozlowska et al., 2017b). When the children were taught to read the somatic signs of HV—what was referred to as their warning signs—and were taught regulation strategies that helped them manage the hyperventilation. they were, in time, able to avert PNES events (Kozlowska et al., 2018b). In children, HV typically occurs in response to current life stress, memories of past stress, or in response to pain.

The second potential mechanism involves stress-related increases is brain norepinephrine. “Norepinephrine (NE) is synthesized in the Locus Coeruleus (LC) of the brain stem, from where it is released by axonal varicosities throughout the brain via volume transmission” (p. 1) (Atzori et al., 2016). Phasic activation of the LC in response to stress results in global activation of the whole brain, shut-down of the prefrontal cortex, and a strengthening of automatic subcortical processes (Arnsten, 2015; Atzori et al., 2016). In adult studies, approximately 70–83% of patients report multiple panic-like symptoms during PNES (Vein et al., 1994; Hendrickson et al., 2014), which is associated with high LC drive and noradrenalin release, as well as enhanced amygdala responsiveness and low prefrontal control (Brehl et al., 2020). It is possible that stress-related surges of norepinephrine contribute to a temporary disruption in horizontal and vertical network function in children with PNES (Barzegaran et al., 2016; Kozlowska et al., 2018a) or to functional shift in organization where functional connectivity patterns that support automatic processes of survival value are prioritized (Arnsten, 2015; Szaflarski and LaFrance, 2018). Norepinephrine surges will both activate brain networks even further and increase the brain’s demand for energy, which the brains of children with PNES—which are already in a state of activation—may or may not be able to meet.

The third potential mechanism is aberrant activation of a range of other neuronal, glial, endocrine, or immune systems and their neurotransmitters, hormones, and signaling molecules (Atzori et al., 2016; Frank et al., 2016). For example, the dissociation literature suggests that states of high arousal may also involve the secretion of endogenous opioids, endogenous cannabinoids, and other anesthetic neurochemicals (Lanius, 2014). Anesthetic neurochemicals are hypothesized to decrease activity in the thalamus and frontal areas (cingulate cortex, orbitofrontal cortex, and insula cortex)—regions that have high levels of opioid receptors (Leppa et al., 2006; Lanius, 2014)—thereby disrupting vertical integration of the brain (Lanius, 2014). Theoretically, interruption of signals from subcortical regions to the cortex could contribute to changes in the level of consciousness and loss of musculoskeletal tone (see above).

Our findings are also interesting to consider from a developmental perspective and in relation to previous EEG studies with adolescents and adults with PNES. Umesh et al. (2017), who used an s-LORETA methodology in a resting-state EEG study with an group of older children—adolescents aged 12–17 years (mean = 15.4 years)—with a slightly longer illness duration (mean = 6 months) than in our study (mean = 4 months) also reported aberrant changes in the gamma band (Umesh et al., 2017). In contrast to our study, however, they found areas of both increased and decreased activation. Xue et al. (2013), using graph theory in a study of adults, found smaller clustering coefficients and global efficiency in the gamma band and decreased long linkage between the frontal region and posterior brain areas compared with controls. It is possible that with time—increased age or illness duration—the pattern of activation/dysregulation changes as a function of allostatic load. The activation of network metrics in the gamma band, as found in the present study, consumes considerable energy and may not be sustainable across time because of energy demands and because of elevated levels of inflammation and oxidative stress (Miller et al., 2018).

Barzegaran et al. (2012) found that PNES correlated with weak local connectedness (≈ local efficiency) and a skewed balance between local and global connectedness (≈ local vs. global efficiency), all in the alpha band. While adult patients did not show activation in network metrics in the gamma band, a number of studies have shown that synchronization between the gamma and alpha bands is important in attention (blocking our distractions), memory processing, and visual and somatosensory processing—all of which can be disrupted in patients with PNES (Kozlowska et al., 2015b; de Vroege et al., 2020). In this context, activation or dysregulation in the gamma or alpha band, or in the pattern of synchrony between them, may reflect related processes—the opposite side of the same coin—that may underpin long-term processing problems. In addition, from a developmental perspective, the findings from Barzegaran et al. (2012) and from the previously discussed studies suggest that with time—age and illness duration—the neural networks of adult patients with PNES become less efficient and more dysregulated. This shift in the pattern of neural markers across development—and the biological cost of allostatic load—parallels the change in various biomarkers in patients with posttraumatic stress disorder (Pervanidou and Chrousos, 2012; Miller et al., 2018) and in individuals exposed to abuse during childhood (Trickett et al., 2010; Bertone-Johnson et al., 2012; Baumeister et al., 2016).

Also of both scientific and clinical importance is the correlation identified between network metrics and arousal: the higher the arousal, the higher the value of the network metric. In current clinical practice, pediatric treatment programs for PNES utilize a range of regulation interventions at multiple system levels: breath-training biofeedback and other bottom-up regulation strategies; regular exercise; trauma-processing interventions; cognitive-behavioral therapy (CBT) and other top-down regulation strategies; pharmacotherapy, and decreasing stress for the child by managing dysfunction in the family system (family therapy). These interventions help regulate and decrease the child’s level of arousal (Kozlowska et al., 2018b, 2020; Gray et al., 2020; Sawchuk et al., 2020). The findings from the current study with children lend support to the proposition that PNES need to be understood more broadly as neural network phenomena (Knyazeva et al., 2011; Barzegaran et al., 2012, 2016; Szaflarski and LaFrance, 2018); this shift in functional organization takes place in the context of increased arousal and is maintained through the flow of energy and matter (Prigogine, 1969; Capra, 1997; Goldbeter, 2018). This loss of healthy equilibrium is associated with an inefficient use of energy, makes neural networks more unstable, and leaves them prone to periods of disorganization, dissolution, or aberrant changes in functional connectivity. Therapeutic interventions that decrease arousal (and energy use) and that help the child regulate in the face of subsequent stress or threat—or perceived stress or threat—will thereby facilitate a shift of neural networks back toward physiological coherence, thereby decreasing the probability of PNES. The developmental perspective suggests that intervention needs to be prompt, facilitating the shift back to physiological coherence before the long-term complications of allostatic load, epigenetic programming, and neuroplasticity make recovery less likely. A number of PNES outcome studies have shown that children with shorter illness duration and acute vs. chronic stress have better outcomes (Yadav et al., 2015; Kozlowska et al., 2018b; Sawchuk et al., 2020).

This study also lends support to the hypothesis that adverse childhood events and maltreatment, in particular, play a role in the neurobiology of PNES. Whist all patients participating in the study reported antecedent stress events, children with PNES reported significantly more maltreatment events than children with other FND symptoms, both on self-report (ELSQ) and on clinical assessment that took place with the child and the family (see Table 3). Maltreatment in childhood is known to upregulate multiple components of the stress system, causing increases in cortisol secretion, autonomic arousal, and inflammation (Trickett et al., 2010; Bertone-Johnson et al., 2012; Michels et al., 2013; Baumeister et al., 2016; Oshri et al., 2020). Because cortisol modulates gene expression in the brain and tissues, the flow-on effects of maltreatment include neuroplasticity changes that change brain structure and function. In a recent neuroimaging–gene expression study in adult patients with FND, Diez et al. (2020) found that physical abuse correlated with increases in functional connectivity between limbic regions and motor cortices (Diez et al., 2020). In the current study, the change in functional organization—an increase in network metrics in the gamma band—arguably reflects a pattern of activation in the face of stress that predisposes children to PNES.

And finally, biological systems have optimal set-points and rhythms—encapsulated in the concept of physiological coherence (McCraty and Shaffer, 2015). While set-points change depending on context and levels of arousal (McEwen, 2000; Hanoch and Vitouch, 2004), the resting-state condition provides an opportunity to assess the system in a safe context that poses no task demands. In this context, while increased network metrics in children with PNES appear to reflect an adaptive response to stress—facilitating local processing of information—failure to return the networks to baseline function in the longer term is likely to be maladaptive. An analogy is found in studies of children with epilepsy, who also show increases in modularity, where repeated activation of neural networks is related to aberrant electrical discharges. In children with epilepsy, increases in modularity over the long term are associated with cognitive impairment and predict illness duration (Vaessen et al., 2013; Paldino et al., 2017). In this way, from a systems perspective, health and well-being are associated with neural network metrics that lie within certain parameters and that maintain certain relationships—reflecting an efficient use of energy and an optimal degree of synchronization between different oscillating systems (McCraty and Shaffer, 2015). By contrast, illness and disease are associated with deviations from these parameters and patterns of synchrony.

This study has five main limitations. First, given that our patient cohort was relatively small—albeit larger than those of previous EEG studies (Knyazeva et al., 2011; Barzegaran et al., 2012, 2016; Xue et al., 2013; Umesh et al., 2017)—our findings will need to be replicated. Second, our study examined neural network function in the resting-state condition. Examination of changes in network metrics immediately prior to onset of PNES, during a PNES episode or during task conditions (e.g., hyperventilation challenge or a stress task) would contribute real-time data pertaining to changes in network dynamics in the context of arousal and stress. Examination of the temporal relationship between changes in network metrics and PNES may be undertaken in future studies. Third, we do not have follow-up network metrics from our cohort following resolution of their PNES and the termination of treatment. In this context we do not know to what degree network metrics normalized following clinical recovery. Fourth, the impact of medication on network metrics could not be assessed due to the small number of children taking medication in the PNES and FND-other groups. Fifth, in the current study we did not undertake a source analysis, which could be the focus of a future study. Notwithstanding the limitations, the current study also has a number of key strengths: the application of graph theory to resting state EEG data in children (not previously done), the presence of two control groups (the FND-other group and healthy controls), and the concurrent acquisition of measures of arousal.

As Keitaro Machida and Katherine Johnson have recently observed, “graph theoretical analysis help[s] to examine how different parts of the brain connect and work together as a whole” (p. 713) (Rubinov and Sporns, 2010; Machida and Johnson, 2019). The results from this study show that children and adolescents with PNES present with brain-wide activation of neural networks (in the gamma band) in the context of high arousal—what we have conceptualized as a state of hypervigilance. The state of hypervigilance confers a survival advantage; it facilitates neural communication and the child’s capacity to respond self-protectively in the face of stressful life events. But the state of hypervigilance also carries a significant biological cost; neural networks are subject to being destabilized in response to perturbations in energy flow or additional demands in the context of new threats, stress, or perceived threats or stress. And in the longer term, the ongoing allostatic processes related to chronically elevated levels of activation, oxidative stress, and inflammation may result in neurobiological consequences that are no longer reversible (Miller et al., 2018).

This study advances the conceptualization of PNES as a stress system disorder—a disorder of neural network dysregulation that manifests in the context of increased brain-body arousal. In this respect, PNES may, in time, be categorized alongside other seizure types: epileptic seizures where network function is disrupted by paroxysmal electrical discharges and where the long-term neuropsychiatric complications arise because of neural network dysregulation (Vaessen et al., 2013; Allebone et al., 2018); seizures secondary to hypoglycemia where network function is disrupted by changes in electrolyte concentrations in neurons and depletion of energy resources (Arieff et al., 1974); and hypoxic seizures where network function is disrupted by lack of oxygen and inability to maintain energy flow (Gastaut, 1974). Moreover, given that PNES occur in the context of high arousal and exposure to adverse childhood experiences, arousal-decreasing interventions on multiple system levels are likely to help the child’s neural networks shift back to physiological coherence. That is, interventions that down-regulate brain-body arousal (and energy use), that increase neurophysiological, emotional, and interpersonal regulation in the face of stress, or that decrease stress in the child family and psychosocial context—should help to reestablish an overall equilibrium characterized by an efficient utilization of energy in the rhythms of daily living (McCraty and Shaffer, 2015). In a state of healthy equilibrium, children would be less vulnerable to neural network dysregulation and instability, and to the clinical manifestation of PNES. Finally, we hope that the present study moves the field ahead by raising important new questions and suggesting directions for future research.

## Data Availability Statement

The data analyzed in this study is subject to the following licenses/restrictions: The ethics and protocol did not include consent for the data set to be made publicly available. Participants and their legal guardians consented for their data to be included into the BRAINnet Foundation’s international brain resource database for reuse when new technologies for analyzing it became available. Requests to access these datasets should be directed to http://www.brainnet.net.

## Ethics Statement

The studies involving human participants were reviewed and approved by the Sydney Children’s Hospital Network Ethics Committee and Sydney West Area Health Service Human Research Ethics Committee. Written informed consent to participate in this study was provided by the participants’ legal guardian/next of kin.

## Author Contributions

KK developed the study protocol and oversaw recruitment and data acquisition. MR and MJ applied graph theory to analyze the EEG data. All the authors contributed to data interpretation and writing of the manuscript.

## Conflict of Interest

The authors declare that the research was conducted in the absence of any commercial or financial relationships that could be construed as a potential conflict of interest.
